# Genome Sequences of Two Marsupial Simplex Viruses, Macropodid Alphaherpesviruses 2 and 4

**DOI:** 10.1128/MRA.01189-20

**Published:** 2021-01-14

**Authors:** P. K. Vaz, T. J. Mahony, C. A. Hartley, J. Motha, J. M. Devlin

**Affiliations:** a Asia-Pacific Centre for Animal Health, Faculty of Veterinary and Agricultural Sciences, The University of Melbourne, Parkville, Victoria, Australia; b Queensland Alliance for Agriculture and Food Innovation, Centre for Animal Science, The University of Queensland, Queensland Biosciences Precinct, St. Lucia, Queensland, Australia; c Agriculture Victoria Research, AgriBio Centre for AgriBioscience, Bundoora, Victoria, Australia; KU Leuven

## Abstract

We present the genome sequences of macropodid alphaherpesviruses 2 and 4, two closely related pathogens of macropods. Both encoded 68 nonredundant open reading frames (ORFs) and share 90.6% genome-wide nucleotide identity. These viruses are associated with fatal outbreaks of disease in multiple marsupial species. These sequences will be important for the development of new diagnostic tools.

## ANNOUNCEMENT

Members of the *Herpesviridae* have linear, double-stranded DNA genomes (120 to 245 kbp in length) and cause significant morbidity and mortality in diverse groups of animals. In marsupials, three alphaherpesviruses are associated with fatal outbreaks of severe respiratory disease and systemic organ failure in both wild and captive populations of macropods. All three cluster within the *Simplexvirus* genus, macropodid alphaherpesviruses (MaHV) 1, 2, and 4 (1–3). MaHV-1 was sequenced in 2016 (4), and here, we report the genome sequences of MaHV-2 and MaHV-4.

The MaHV-2 and MaHV-4 isolates sequenced in this report were originally isolated from diverse macropod species showing severe clinical signs of disease. MaHV-2 (strain V3077/08) was isolated from a quokka (Setonix brachyurus) and a dorcopsis wallaby (Dorcopsis luctuosa) in 1978 (2) and MaHV-4 (strain V3116/09) from a free-ranging eastern gray kangaroo (Macropus giganteus) in 2009 (3). For this report, viruses were propagated in JU56 wallaby fibroblast cells (5), and DNA was purified from herpesvirus nucleocapsid preparations (6). Library preparations used 50 ng of viral genomic DNA using the Illumina Nextera DNA library preparation kits according to the manufacturer’s instructions and were loaded onto an Illumina MiSeq instrument. Sequencing was carried out using a 300-cycle v2 sequencing-by-synthesis (SBS) kit (Illumina, Inc.) in paired-end 150-bp format (see Table 1 for sequencing details). The adaptors were removed and the reads filtered based on Phred20 with Trim Galore v0.6.5, and the genomes were *de novo* assembled using SPAdes v3.12.0 with default parameters on careful mode; in addition, *de novo* assembly was performed using medium-low default sensitivity settings on the bioinformatics package Geneious v6.1.8 (4). For each virus species, this initially yielded large contigs with consensus sequences that corresponded to herpesvirus sequences, with high similarity to the MaHV-1 genome and to published fragments of MaHV-2 and MaHV-4 sequences. These contigs were then used as references in further assemblies, where reads were reiteratively mapped until there was no further contig extension. Where available, prior information about the viral genomic structure was utilized to assist in the final assemblies.

**TABLE 1:** Summary of viral genomes

Statistic	Data for:	
	MaHV-2	MaHV-4
Genome size (kbp)	142	141^*a*^
Structure	Type E	Unknown
No. of reads (mapped)	2.1 (1.7) million	3 (2.1) million
Coverage (avg no. of reads/bp)	1,576	1,985
Unique long region size (kbp)	97.8	97.9
Unique short region size (kbp)	15.5	15.3
Total size of repeat regions (kbp)	14.2	13.7^*b*^
Inverted repeat short region size (kbp)	7.3	ND^*c*^
Inverted repeat long region size (kbp)	6.9	ND
Total no. of ORFs (unique)	68	68
% GC content	52.6	51.5
GenBank accession no.	MT900475	MT900474
SRA accession no.	SRR12450755	SRR12450754

a Estimated based on the size of the unique regions and the sum of the repeat regions.

b As the genomic structure is unknown, only the cumulative repeat region is reported.

c ND, not determined.

The structure of the MaHV-2 genome was previously determined in 1987, using restriction enzyme (RE)-Southern blot hybridization mapping analysis, to have a type E structure typical of a simplexvirus and to occur as four equimolar isomeric genome arrangements (7). Type E genomes consist of a unique long (UL) region flanked by large inverted repeats (RL), and a unique short (US) region, flanked by another set of large inverted repeats (RS). Using RE-Southern blot analysis, they estimated the genome to be approximately 135 kbp in length, consisting of a 95-kbp UL region, a 5.5-kbp RL region, a 15-kbp US region, and a 7-kbp RS region. Our sequencing analysis of MaHV-2 was largely consistent with these earlier estimations, although we determined that MaHV-2 had a slightly larger genome size of approximately 142 kbp, a UL region of 97.8 kbp flanked by 6.9-kbp (RL) inverted repeats, and a US region of 15.5 kbp flanked by 7.3-kbp (RS) inverted repeats. The final structure of the MaHV-4 genome has not been determined as we were unable to resolve the genome termini. However, based on the sequence similarity to MaHV-2, we estimate that it is also likely to be a type E structure typical of simplexviruses. The core genome was determined to be approximately 127 kbp (excluding terminal repeats that are estimated at 13.7 kbp combined), consisting of a UL region of 97.9 kbp, a US region of 15.3 kbp, and a 13.7-kbp internal repeat region. The final genome size was estimated to be approximately 141 kbp, with the inclusion of terminal repeats. The MaHV-2 and MaHV-4 genomes share 90.6% nucleotide pairwise identity and share 70.4% and 71.3% identity to that of MaHV-1, respectively.

Open reading frame (ORF) detection was performed using Glimmer3, with annotation nomenclature following that of MaHV-1 (GenBank accession number NC_029132.1). Small sections of the genome were excluded from ORF detection, specifically, regions of low sequence confidence, possibly sites where genomic DNA breakage occurred during library preparation. Additionally, these specifically corresponded to regions where the large inverted repeats (RS and RL) joined to the unique regions (UL and US) and so are sites where genome isomeric inversions may occur (7), disrupting sequencing readthrough and consensus. Both viruses encode 68 distinct ORFs, including many that are common to eutherian herpesviruses. However, similarly to MaHV-1, both MaHV-2 and MaHV-4 lacked four ORFs typically conserved in eutherian and avian alphaherpesviruses, specifically, UL3, UL4, UL56, and glycoprotein J.

MaHV-2 and MaHV-4 both share several unique ORFs with MaHV-1, including PW3-PW6, although their functions remain uncharacterized. The absence of several alphaherpesvirus-conserved genes, as well as the shared presence of MaHV-unique ORFs, indicate that these changes occurred prior to viral speciation of the macropodid alphaherpesviruses from a common ancestor.

The genome sequences of MaHV-2 and MaHV-4 will be valuable for diagnostic purposes and the further development of new tools that could be used in the management of disease outbreaks.

### Data availability.

The genome sequences and read data sets have been deposited at GenBank and can be found under BioProject accession number PRJNA656846 and GenBank accession numbers MT900475 (MaHV-2) and MT900474 (MaHV-4).
